# Continuous radon monitoring during seven years of volcanic unrest at Campi Flegrei caldera (Italy)

**DOI:** 10.1038/s41598-020-66590-w

**Published:** 2020-06-12

**Authors:** C. Sabbarese, F. Ambrosino, G. Chiodini, F. Giudicepietro, G. Macedonio, S. Caliro, W. De Cesare, F. Bianco, M. Pugliese, V. Roca

**Affiliations:** 1 Dipartimento di Matematica e Fisica, Università degli studi della Campania “L. Vanvitelli”, viale Lincoln 5, 81100 Caserta, Italia; 2 Istituto Nazionale di Fisica Nucleare, sezione di Napoli, via Cintia 21, 80126 Napoli, Italia; 3 Istituto Nazionale di Geofisica e Vulcanologia, sezione di Bologna, via D. Creti 12, 40124 Bologna, Italia; 4 Istituto Nazionale di Geofisica e Vulcanologia, Osservatorio Vesuviano, via Diocleziano 328, 80124 Napoli, Italia; 5 Dipartimento di Fisica, Università degli studi di Napoli “Federico II”, Via Cintia 21, 80126 Napoli, Italia

**Keywords:** Natural hazards, Geochemistry

## Abstract

This is a seven-year study (1/7/2011-31/12/2017) of radon monitoring at two sites of Campi Flegrei caldera (Neaples, Southern Italy) that in the last 70 years experienced repeated phases of volcanic unrest. The sites are equipped with devices for radon detection, based on the spectrometry analysis of the α-particles of radon daughters. A hybrid method, as combination of three known methods, is applied for the identification of residuals (anomalies) and trends of the time series of Radon. The results are compared with the following indicators of current caldera unrest: the tremor caused by the major fumarolic vent registered by a seismic station; the cumulative of background seismicity; the maximum vertical deformation acquired by GPS networks during the current phase of uplift; the temperature-pressure of the hydrothermal system estimated based on gas geo-indicators. The comparisons show strong correlation among independent signals and suggest that the extension of the area affected by current Campi Flegrei crisis is larger than the area of seismicity and of intense hydrothermal activity from which the radon stations are 1–4 km away. These results represent an absolute novelty in the study of a such calderic area and mark a significant step forward in the use and interpretation of the radon signal.

## Introduction

Radon (^222^Rn) is a radioactive element with a half-life of 3.82 days, formed in the Earth’s Crust by the radioactive decay of ^226^Ra in the ^238^U decay series. Faults and fractures are preferential migration pathways for radon gas and carrier gases (CO_2_), from the deep layers of the Earth’s Crust to the surface^1–3^. The radon signal monitored in soils contains both local and remote information and is influenced by many environmental factors^4–7^. It is well established that radon can provide useful information if it is continuously recorded at a given site. The continuous monitoring allows us to characterize the local background signal and recognize the complete trends of local and remote effects on the signal^8,9^. The local effects due to sudden changes in temperature, pressure or water content in the soil (heavy rain or no rain for long periods) can be so predominant and often create large variations in the signal^10–12^ to hide components due to remote geophysical sources^5,6,13–16^.

In recent years, interest of the international scientific community towards the study of radon emission as a tracer of natural endogenous phenomena (seismic and volcanic activity) has considerably grown^17–33^.

**Figure 1:**
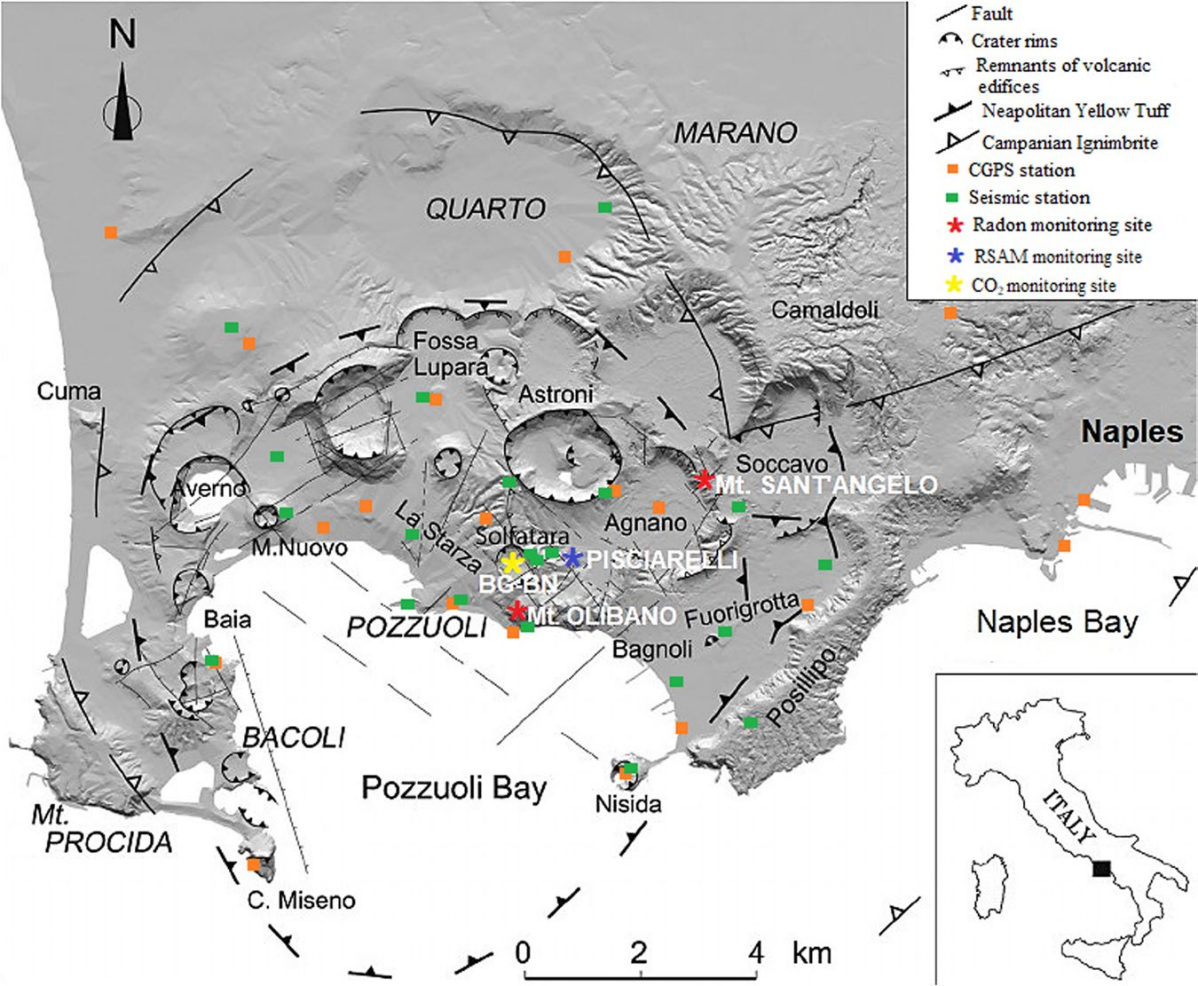
Map of the Campi Flegrei caldera (Naples-Italy). The map, modified after^16^ (10.1016/j.apradiso.2020.109140) using the PAINT (Microsoft Corporation, Version: 6.1907.18017.0), shows the structural setting of the caldera characterised by tectonics and volcano-tectonic activity. The two radon monitoring sites of Monte Olibano and Monte Sant’Angelo, and the others monitoring sites of geochemical and geophysical parameters are reported. The squares represent the Campi Flegrei seismic network in green and the NeVoCGPS network in orange (see Methods).

The present study focuses on the analysis of radon activity concentration time series over a period of about seven years, from 1 July 2011 until 31 December 2017, obtained by continuous monitoring in soils at two sites of the Campi Flegrei caldera (Naples, Italy). The two sites are located in Monte Olibano, in the central area of the caldera, and Monte Sant’Angelo, where the Department of Physics ‘E. Pancini’ of the University of Naples “Federico II” is located (Fig. 1).

Campi Flegrei is a caldera of about 120 km^2^ located on a NE–SW-trending structure in the Campanian Plain. The caldera formed from the collapses associated to the large size eruptions of the Campanian Ignimbrite and of the Neapolitan Yellow Tuff^34–36^ (Fig. 1). The structural setting of Campi Flegrei is characterised by NE-SW and NW-SE trending faults that control the distribution of volcanic centres. Since the middle of the 20^th^ century, the caldera has been subjected to a long-term crisis characterized by numerous episodes of ground uplift and correspondent seismic activity with a maximum total ground uplift of about 4 m in 1983–1984^37^. After that period the caldera subsided until 2004–2005 when a new uplifting phase started. In the 2012–2013 period, there was an increase in the acceleration of the deformation of the soil in the caldera area^38,39^, interpreted as the effect of the intrusion at shallow depth^38^ of magma within a sill-like structure.

This renewed uplift is still ongoing and is accompanied by large variation in the degassing rate and fumarolic composition at Solfatara and by earthquakes mostly located at shallow depth (<2 km) in the subsoil of Solfatara^40–44^. The caldera is characterized by the emission of hydrothermal-volcanic fluids from both fumarolic vents, with temperature as high as 160–165 °C, and process of soil diffuse degassing. The associated deeply derived CO_2_ fluxes are considerably high being estimated in the range 1000–3000 tons/day^40–42^. The emission of geothermal fluids, both through diffuse degassing and vents, dominate the present-day Campi Flegrei caldera energy budget with the emission of several kilotons of vapour every day and with a cumulative thermal energy output of about 5 ∙ 10^16^ J^45^.

Here, the time series analysis of the radon signal was performed using a well-proven hybrid method that has successfully been applied to the identification of anomalies and trends in time series^8,9,25,46–49^. The hybrid method is formed by sequential aggregation of: (i) the Multiple Linear Regression (MLR) for filtering the known modulations out from the time series, (ii) the Empirical Mode Decomposition (EMD) techniques for the signal decomposition, and (iii) the Support Vector Regression (SVR) technique for forecasting the time series. The forecasting results are then used in comparison with raw time series in order to identify the anomalies (see Methods)^8,9,25^. The aim of this study is to investigate the trends and anomalies in the radon signals in comparison with other geo-indicators of the caldera unrest: seismicity, ground deformation^44^ and, for the first time in the literature, the seismic tremor of the main fumarole that has been identified as a powerful indicator of the changes affecting the hydrothermal system^41,50^.

## Results

Our analysis of the radon signal was focused on distinguishing and extracting the desired information including the estimation of the different components and trends and the recognition of the possible anomalies^46,47,51,52^. The analysis was performed using a hybrid method that aggregates multiple separate methods and combines the advantages of the single ones with optimized algorithms^48,49,52–54^, which are described in the Methods section.

**Figure 2:**
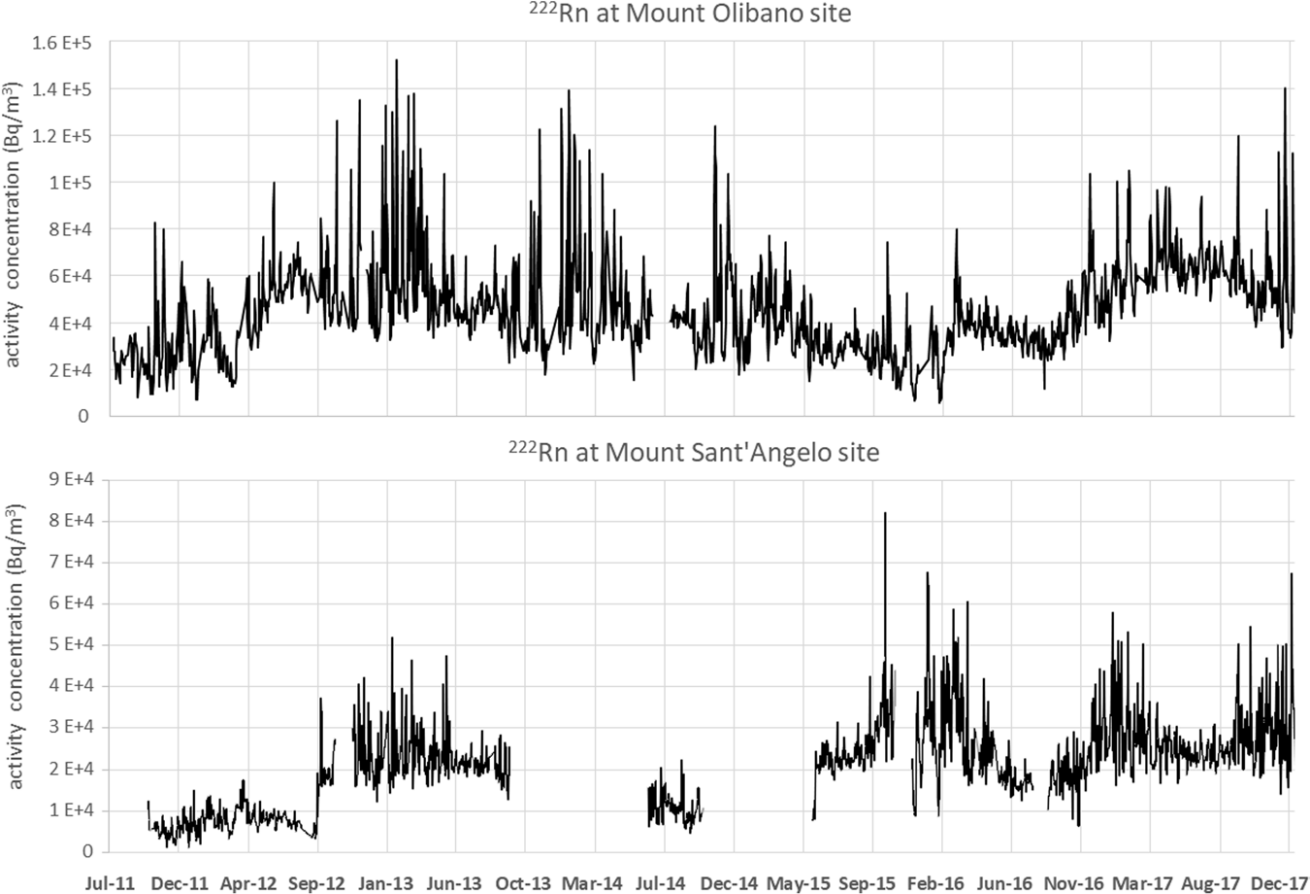
Radon time series recorded at OLB and MSA sites. The two series of radon activity concentrations (Bq/m^3^), corrected for meteorological influences with the MLR method, recorded in the two monitoring sites at Monte Olibano (**a**) and Monte Sant’Angelo (**b**) within the caldera of Campi Flegrei, are shown. Some lack of data is due to the failure of the data logging network.

The two daily time series (1/7/2011-31/12/2017) of ^222^Rn activity concentration in soils recorded at Monte Olibano (OLB) and Monte Sant’Angelo (MSA) stations are displayed in Fig. 2. The signals were corrected for the recorded influencing outside driving forces, i.e. temperature, relative humidity and pressure, through MLR method^8,9^.

The ^222^Rn signals were investigated with the Empirical Mode Decomposition (EMD, see Methods), a method not significantly affected by the lack of data in the time series thanks to self-consistent regression estimator^53^. Most of the components obtained by applying the EMD method (not showed for brevity) are due to meteorological and seasonal phenomena.

We focus here on the trends and the residuals of the two signals. The trend is the last component obtained by the EMD decomposition method^25,54^ while the residuals (i.e. anomalies) refer to those values of the radon time series that are out of the 95% confidence interval of the differences between the measured signals and the forecasted time series. The forecasting of the two radon time series is obtained by the application of the forecasting Support Vector Regression (SVR) method^8,9,49^ on the components obtained from MLR + EMD hybrid method, and hence on their sum (see Methods).

**Figure 3:**
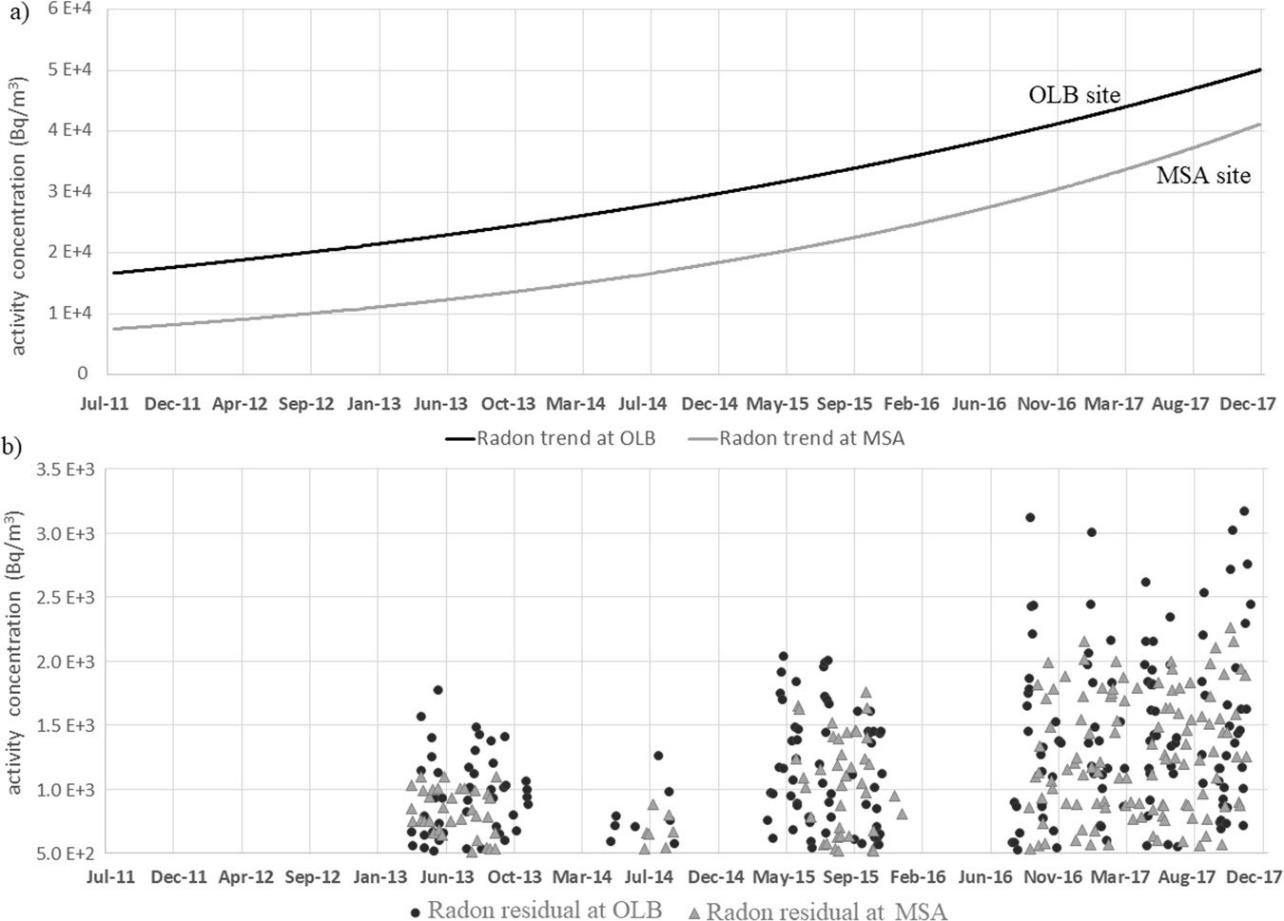
Radon trends and residuals. (**a**) Radon trends at OLB and MSA sites, obtained by applying the MLR + EMD hybrid method. (**b**) Radon residuals in OLB and MSA sites, obtained by subtracting the computed signals, by using the hybrid method MLR + EMD + SVR, from the raw recorded data, within a 95% CI (see section Time series analysis).

The trends (Fig. 3a) reflect an increasing pattern of ^222^Rn activity concentration during the entire monitoring period at both OLB and MSA sites. Instead, radon residuals concentrate in 4 well defined periods (March-November 2013, May-September 2014, April-November 2015 and August 2016-Decemner 2017; Fig. 3b). It is worth noting how the radon residuals occur almost contemporaneously at OLB and MSA, two sites 3.87 km apart, even if with different intensity possibly due to the different distance from the centre of the current unrest that roughly coincides with the central area of the caldera where the hydrothermal activity is concentrated and where the maximum ground uplift and the highest seismicity are recorded.

## Discussion

**Figure 4:**
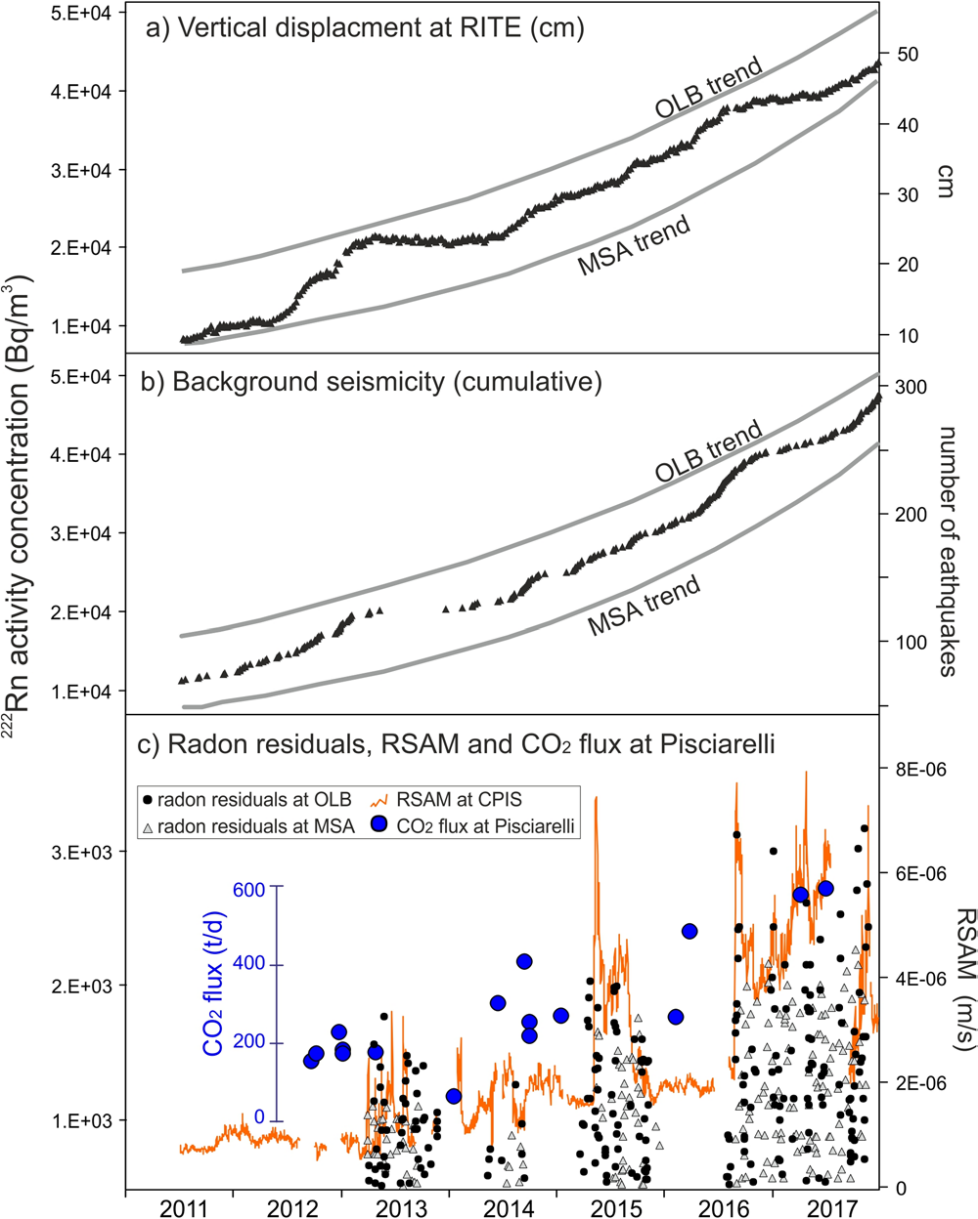
Comparison of the radon trends and residuals with independent geophysical signals. Radon trends at OLB and MSA sites compared with (**a**) maximum vertical displacement at Campi Flegrei (RITE continuous GPS station^45^) and (**b**) background seismicity (i.e. the cumulative number of days with earthquakes since 2000^43^). (**c**) Radon residuals, RSAM measured by CPIS station close to Pisciarelli fumarolic vent^41^, and CO_2_ flux from Pisciarelli vent.

The radon signals, monitored during 2011–2017, refer to the current period of Campi Flegrei volcanic unrest (post 2004–2005 period) characterised by soil uplift, increasing seismic activity and marked variation in the hydrothermal activity. The trends and residuals obtained from the radon signals monitored in soils of OLB and MSA sites are thus comp ared with independent data that were recently used to characterise the current phase of Campi Flegrei volcanic unrest (Fig. 4a,b). In particular we considered the following data sets:

- the ground deformation data of Campi Flegrei area^45^ (Fig. 1, see Methods) synthetically represented by the vertical displacement at RITE GPS station where the maximum uplift values are recorded;
- the cumulative number of days with earthquakes since 2000, i.e. the background seismicity as defined in ref. ^45^;
- the fumarolic tremor (RSAM) of Pisciarelli fumarole^50^ registered by the CPIS seismic station (40°49'45.8” N, 14°08'49.9” E).

Fumaroles are in fact known to generate seismic (and infrasonic) tremor, whose temporal changes quantitatively correlates with the variations of the fumarole activity and ground shaking^55,56^. In particular, the RSAM at Pisciarelli was recognised as a powerful indicator of the current unrest of Campi Flegrei^41,50^. The RSAM is in fact controlled by the gas fluxes from the fumarolic vent (currently the main vent of Campi Flegrei) as suggested by the high correlations with both direct flux measurements and the CO_2_ air concentration continuously measured at the site (see Fig. 11 in ref. ^41^).

The correlation of the radon trends with ground deformation (Fig. 4a) and the background seismicity (Fig. 4b) suggests in general the high potentiality of the radon as an additional indicator of the current crisis of Campi Flegrei. The increased permeability of the media due to the increased number of earthquakes can significantly contribute to outgassing variations^57,58^ and ultimately to the increase of the radon trends. However, the MSA radon station is ~ 4 km distant from the seismic zone that is currently restricted to the Solfatara and Pisciarelli hydrothermal sites (see Fig. 1 in ref. ^45^). Also, for this reason, we think that a general increase of the temperature and the pressure of the Campi Flegrei hydrothermal system^50,59^ is the central parameter explaining the correlation among the different independent variables. The temperature increase may induce thermal devolatilization phenomena on radon^60^ and the fluid pressure increase favours deformation, earthquakes and the flux of the hydrothermal gas from depth to the surface. For example, it was measured by various authors a strong increase in the hydrothermal-volcanic CO_2_ both diffusively emitted at Solfatara (from 900–1000 t/d in 2010–2011 to 1500–3000 t/d in 2015–2016^43^) and emitted by Pisciarelli vent (from 150–200 t/d in 2012–2013 to 400–600 t/d in 2017–2019^44^, Fig. 4c).

In this context, it is particularly indicative the correlation between the radon residuals and the RSAM (Fig. 4c), a signal directly controlled by the Pisciarelli emission. The radon residuals mimic the prominent fumarolic tremor enhancement peaks during the entire monitoring time because the four periods of high radon residuals practically coincide with the periods of highest seismic tremor of the hydrothermal area.

This evident correlation proves that, independently from its generation-propagation mechanisms, the RSAM signal is actually linked to dynamic changes in gas flow regime within the Campi Flegrei caldera system. In particular, the 2012 to 2017 increasing CO_2_ emission of Pisciarelli vent^44^ testifies a general increase of the fluid pressure of the Campi Flegrei hydrothermal system and consequently in the hydrothermal CO_2_ fluxes affecting the caldera, a process that in some way can control the ^222^Rn increasing trends and residuals.

The carrier gases, like CO_2_, play, in fact, a critical role in controlling the migration and transport of trace gases such as ^222^Rn towards the surface^1,17,51,61–64^. Due to the diffusion coefficient in dry soil (5 ∙ 10^−6^ m^2^/s)^65^ and half-life (3.82 days), the large ranges of soil ^222^Rn activities can be explained by a double origin^1^: (i) the radon is generated by the decay of ^238^U accumulated in the soil (usually low activity concentration of several Bq/m^3^); (ii) the radon produced by the decay of ^238^U from deeper levels migrates upward, transferred by carrier gases through faults and fractures (high activity concentration up to several ten thousand Bq/m^3^). The OLB and MSA data (^222^Rn activity concentrations of 10^4^–10^5^ Bq/m^3^) point to this second type of origin and their trends and residuals, well correlated with independent geophysical data, are well explained by variation in the flux of the hydrothermal-volcanic CO_2_ that at Campi Flegrei acts as carrier gas.

The different intensities of radon trend curves and residuals, lower in MSA than in OLB, are likely due to the different distance of the two sites from the main active degassing area, i.e. Pisciarelli fumarole and Solfatara crater, where the CO_2_ flux is higher. Furthermore, OLB is inside the area of current seismicity, where gas fluxes can be increased also by earthquake induced fracturing. Hence, the lower intensity of radon trend and residuals at MSA with respect to OLB, is most likely due to a lower carrier gas effect.

**Figure 5:**
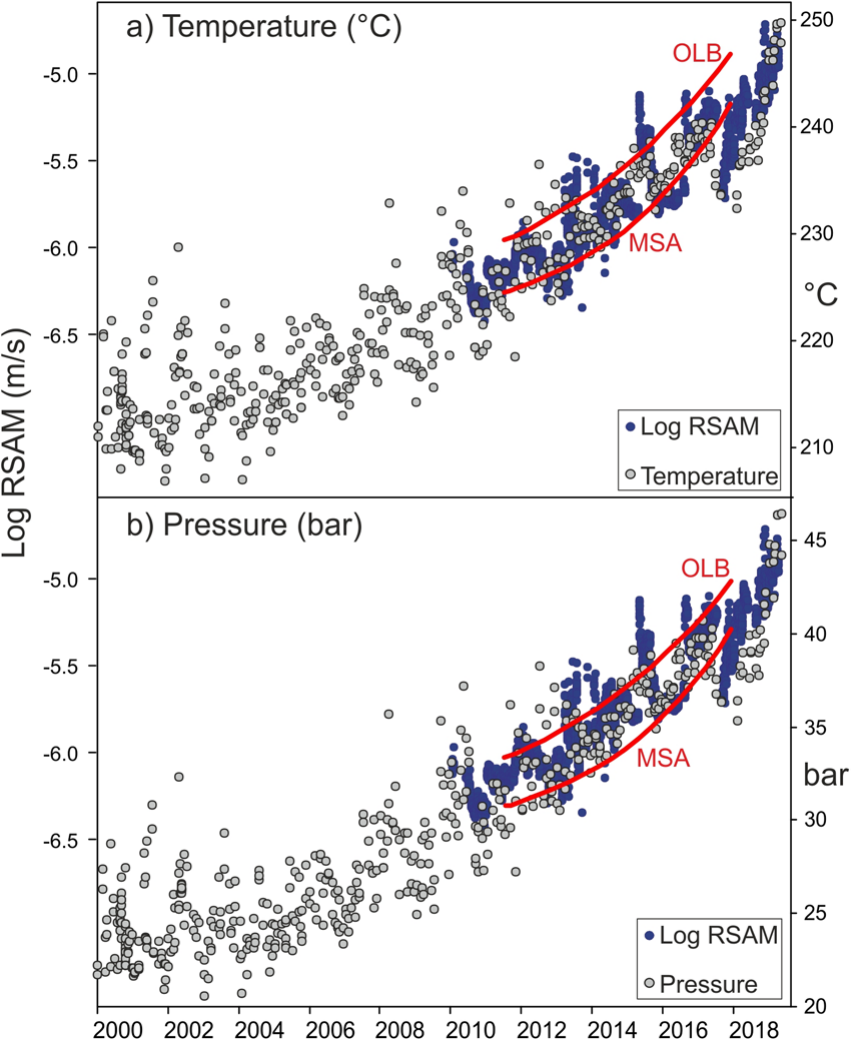
Comparison of temperature and pressure with radon trends and RSAM. Temperature (**a**) and pressure (**b**) hydrothermal system estimated by applying a gas-equilibria approach applied to the hottest fumaroles of Campi Flegrei. See refs. ^45,50^ for further details on the computations. The T-P estimates are compared with the log RSAM at CPIS^41,50^. The ^222^Rn trends returned by EMD for OLB and MSA are also reported with the red line (see Fig. 3a for the scale).

All the signals that we have discussed are thus likely controlled by the temperature-pressure of the hydrothermal system whose increase strongly characterises the activity of the Campi Flegrei in these early decades of the XXI century (Fig. 5). The T-P of the hydrothermal system, estimated by applying a gas-equilibria approach to the fumaroles of highest temperature^45,50,66^ (BG and BN, ~160 °C and ~145 °C, respectively; Fig. 1), started to increase since the beginning of 2000’s in a process that is still ongoing (Fig. 5).

## Conclusions

Since 2004–2005 Campi Flegrei caldera is in a new unrest period characterised by ground uplift, seismicity, changes on fumarolic fluid composition and a general increase of the emission of volcanic-hydrothermal fluids. The long data series, acquired since 2011, highlight how this crisis in some way affects also the ^222^Rn activity in the soils. The data suggest two main considerations: (i) the first regards the extension of the area affected by the current unrest that results much larger than the area of seismicity and the area of intense hydrothermal activity of Pisciarelli and Solfatara (e.g. MSA site is in Napoli city, 4 km east from the centre of the crisis); (ii) the second consideration regards the ^222^Rn and its potential as an indicator of the evolution of a volcanically-induced crisis: the radon signals show, in fact, a pattern very similar to that of the more classic geophysical and geochemical parameters routinely monitored for the surveillance of volcanic systems. These results represent an absolute novelty in the study of a such calderic area and mark a significant step forward in the use and interpretation of the radon signal, and also indicate that long-term observation of the radon signal at multiple sites in a seismic-volcanic area could help improve the characterization of the region.

## Methods

### Monitoring data

^222^Rn activity concentration (Bq/m^3^) in soil gas of Campi Flegrei caldera is continuously monitored, at Monte Olibano and Monte Sant’Angelo sites (Fig. 1), by RaMonA system^67,68^. It is based on electrostatic collection of the ionized descendants of the gas on a Silicon detector inside a cylindrical metallic chamber and provides the complete alpha spectrum of ^220^Rn and ^222^Rn that is analysed by FORTAS software^67^. Here, only ^222^Rn is considered, although ^220^Rn could be used to discriminate the source coming from deeper levels^8^. The continuous monitoring is performed by 1 l/min pumping the soil gas from a depth of 0.80 m to the inlet of the detector^19^. The time interval of detected and processed data is 1 h, but the daily average of the data is used in the analysis. The device also monitors temperature, relative humidity and pressure inside the detection chamber and in the surface soil of the measurement site. The values of the climatic parameters measured internally are used to correct the rough result on the basis of the variability of the detection efficiency^7^; while externally measured values are used to search for correlations with radon through the MLR method^9,19^.

The seismic tremor is monitored from a station located 8 m away from the Pisciarelli fumarole (Fig. 1), which measures changes in ground vibration, in m/s, in real time (real-time seismic amplitude measurement - RSAM). Analytical methodologies and uncertainties are described in refs. ^50,69^. A characteristic RSAM value is computed daily by assuming as representative the minimum value registered during the night, which corresponds to the time of the day when anthropogenic noise is lowest (lower than during daylight hours).

The chemical composition (μmol/mol) of Solfatara fumaroles (Bocca Grande, Bocca Nuova) is monitored by systematic sampling (Fig. 1). The analytical methods and their uncertainties are available in the refs. ^64,70^. The time series of the CO/CO_2_ ratio is calculated since it is an excellent indicator of the temperature variations at depth^42^.

The background seismicity is referred to the cumulative distribution of Campi Flegrei seismicity, which simply corresponds to the sum of the days in which at least one earthquake has occurred^43^. The current permanent seismic network of Campi Flegrei (Fig. 1) is composed of a total of 23 stations that transmit data in real time to the Osservatorio Vesuviano (Istituto Nazionale di Geofisica e Vulcanologia) monitoring center. The reference station for Campi Flegrei seismicity is located close to the Pisciarelli hydrothermal area where the post-2000 seismicity is concentrated. A full description of Campi Flegrei network is reported in ref. ^45^.

The ground deformation (cm) is monitored through the NeVoCGPS (Neapolitan Volcanoes Continuous GPS) network (Fig. 1), which provides measurements of the 3D time changes in 20 stations operating at Campi Flegrei^45^. A full description of NeVoCGPS network is reported in refs. ^71,72^. The reference GPS station for the Campi Flegrei area is close to the zone of maximum vertical displacement, near the Solfatara crater, because it is representative of the time pattern of ground deformations.

### Time series analysis

Hybrid methods are an effective methodology that can aggregate multiple separate methods, combining the advantages of each one with optimized algorithms and achieving greater accuracy since the absolute uncertainty is lower than that obtained by every single method^48,49,54,73^.

The hybrid method Multiple Linear Regression + Empirical Mode Decomposition + Support Vector Regression (MLR + EMD + SVR) used for radon time series analysis, to obtain the trends and anomalies detection, is here developed and tuned in MATLAB environment. This appropriate combination coming from previous works^8,9,25,73–75^ that studied the analytical performance and advantages of each component method, tested on several time series, in order to obtain the best performance with lower uncertainty. The signal trend extraction is performed by using the MLR + EMD method. The MLR technique estimates the contribution of the recorded environmental parameters (i.e. temperature, relative humidity and pressure, which influence the signal) to the time series, via a multiple linear regression model based on the least-squares fit. The final correct time series are obtained as the difference between the unprocessed time series and that obtained from the model. The procedure is fully described in refs. ^19,46^. On the correct time series the EMD technique is applied to further differentiate the seasonal-periodic modulations within the signal, by decomposition. The signal is decomposed into a collection of components, progressively in frequency (unless constancy or monotony), obtained by iterative differences between the signal and the mean envelope of the upper and lower envelopes from the spline interpolation among all the local maxima and the local minima, respectively. The last component is the representative trend of the time-series. The detailed analytical description is reported in refs. ^76,77^. The EMD method is improved to deal with time series with missing data^78^: self-consistent regression estimator recursively imputes missing values and decomposes the signal efficiently under EMD framework^47^. The anomalies identification, which underlines the specific values not attributable to the normal evolution of the studied time series, is performed by observing the raw signal outside the two 95% confidence bounds of the forecasted signal obtained using the SVR technique combined to the output of MLR + EMD hybrid method. The SVR method makes a regression model based on a set of non-linear training data, in order to predict them with a function (i) having lowest deviation from the original data set and (ii) being as flat as possible. This regression problem is commonly used in its Lagrange dual formulation: it allows to extend from non-linear functions into a high-dimension dual space where linear techniques are available, using a Gaussian kernel mapping function. The SVR method is here applied first on each output component from MLR + EMD hybrid method, and finally on the sum of all predicted components; this is the expected forecasting result of the raw time series. A complete analytical description of the forecasting process used by the SVR method is available in refs. ^73,79^, with numerous explanatory examples. A datum in the raw time series is considered an anomaly if it is beyond one of the two forecasted time series at 95% CI that contain the same raw signal: then, the residuals (i.e. anomalies values) are obtained by subtracting the two forecasted signals at 95% CI from the raw time series, in absolute terms.

**Figure 6:**
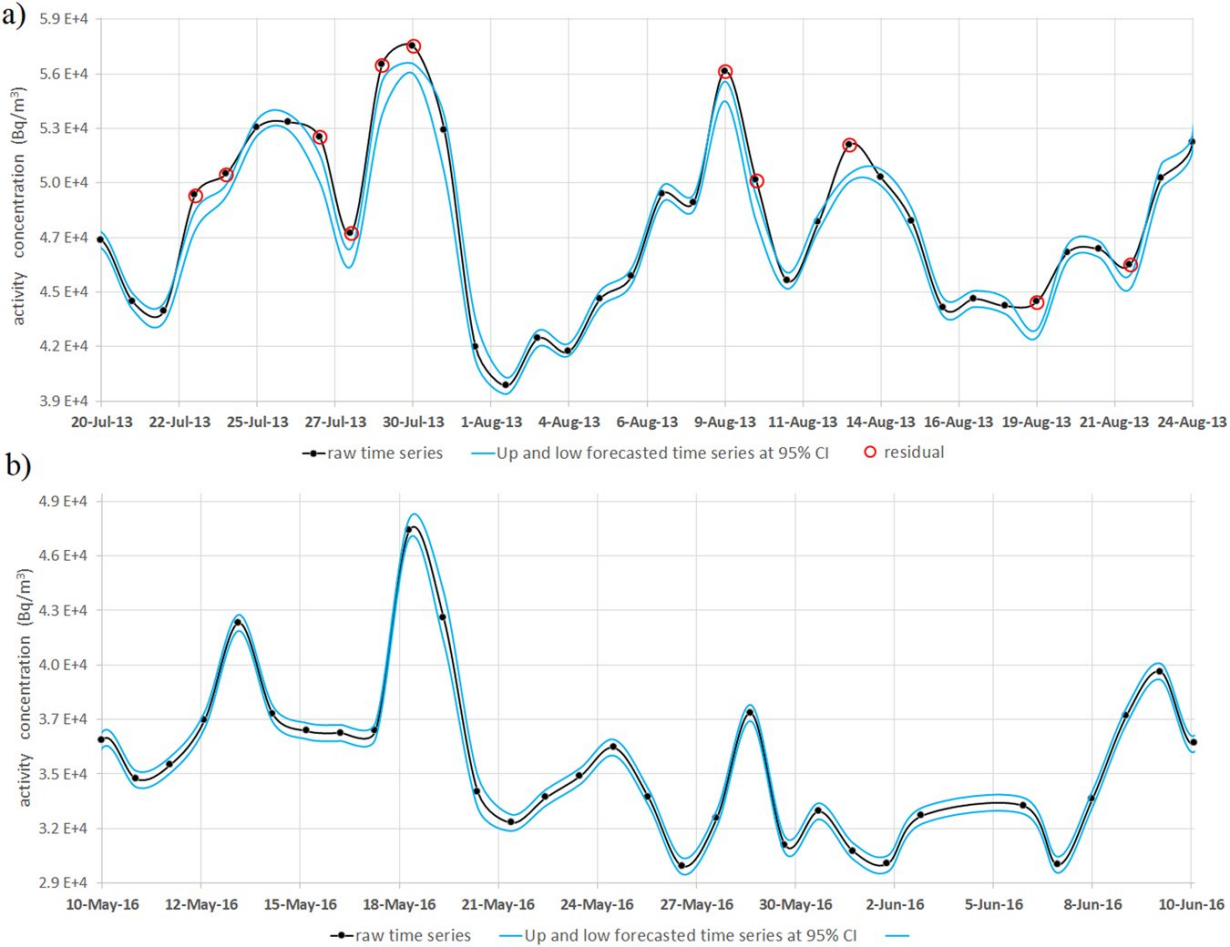
Example of residuals detection criterion applied two parts of the radon time series at OLB site. The graphs show the raw signal of the ^222^Rn activity concentration at Monte Olibano (in black) site during the period 20 July 2013–24 August 2013 (**a**) and the period 10 May-10 June 2016 (**b**), delimited by two signals (in cyan) obtained by the forecasted time series, from MLR + EMD + SVR method, at 95% CI. The data that are beyond those two levels, present only in the graph (**a**), are denoted as residuals (red circles). Hence, the residuals values are obtained as difference between the raw signal amplitude and one of two levels (lower or higher).

The two forecasted time series at 95% CI are calculated as the forecasted signal results from SVR method ± the confidence coefficient 1.96 multiplied by the mean squared errors of the forecasting. To better understand the anomalies detection, two examples are shown in Fig. 6 where two parts of the ^222^Rn activity concentration time series of Monte Olibano are analysed: one with (20 July-24 August 2013) and another without anomalies (10 May-10 June 2016). The anomalies identification process is reported in refs. ^74,80^.

## Supplementary information

Supplementary Information.

Supplementary Information 2.

## Data Availability

All the recorded data (Radon, temperature, humidity and atmospheric pressure) their graphs are reported in the Supplementary Material.
