# Supplementary material for: Continuous radon monitoring during seven years of volcanic unrest at Campi Flegrei caldera (Italy)
Source: Sci Rep. 2020 Jun 12;10:9551. doi: 10.1038/s41598-020-66590-w (PMC7293226; doi:10.1038/s41598-020-66590-w)
Supplement: Supplementary file 1 — Supplementary Information. [file 41598_2020_66590_MOESM1_ESM.docx]

Supplementary Information for

**Continuous radon monitoring during seven years of volcanic unrest at Campi Flegrei caldera (Italy)**

C. Sabbarese^1,2,*^, F. Ambrosino^1,2,^, G. Chiodini^3^, F. Giudicepietro^4^, G. Macedonio^4^, S. Caliro^4^, W. De Cesare^4^, F. Bianco^4^, M. Pugliese^5,2^, V. Roca^2^

^1^ Dipartimento di Matematica e Fisica, Università degli studi della Campania “L. Vanvitelli”, viale Lincoln 5, 81100, Caserta, Italia.

^2^ Istituto Nazionale di Fisica Nucleare, sezione di Napoli, via Cintia 21, 80126, Napoli, Italia.

^3^ Istituto Nazionale di Geofisica e Vulcanologia, sezione di Bologna, via D. Creti 12, 40124 Bologna, Italia.

^4^ Istituto Nazionale di Geofisica e Vulcanologia, Osservatorio Vesuviano, via Diocleziano 328, 80124, Napoli, Italia.

^5^ Dipartimento di Fisica, Università degli studi di Napoli “Federico II”, Via Cintia 21, 80126 Napoli, Italia

^*^ Corresponding author: Carlo Sabbarese

email: [carlo.sabbarese@unicampania.it](mailto:carlo.sabbarese@unicampania.it)

The attached PDF file named “Supplementary material Sabbarese et al.xlsx” includes:

All the recorded data in two sites (Radon, temperature, humidity and atmospheric pressure) and their graphs.
